# Graded fMRI Neurofeedback Training of Motor Imagery in Middle Cerebral Artery Stroke Patients: A Preregistered Proof-of-Concept Study

**DOI:** 10.3389/fnhum.2020.00226

**Published:** 2020-07-14

**Authors:** David M. A. Mehler, Angharad N. Williams, Joseph R. Whittaker, Florian Krause, Michael Lührs, Stefanie Kunas, Richard G. Wise, Hamsaraj G. M. Shetty, Duncan L. Turner, David E. J. Linden

**Affiliations:** ^1^School of Psychology, Cardiff University Brain Research Imaging Centre (CUBRIC), Cardiff, United Kingdom; ^2^Department of Psychiatry, University of Münster, Münster, Germany; ^3^Max Planck Adaptive Memory Research Group, Max Planck Institute for Human Cognitive and Brain Sciences, Leipzig, Germany; ^4^School of Physics and Astronomy, Cardiff University, Cardiff, United Kingdom; ^5^Department of Cognitive Neuroscience, Maastricht University, Maastricht, Netherlands; ^6^Department of Cognitive Neuroscience, Donders Institute for Brain, Cognition and Behaviour, Radboud University Medical Center, Nijmegen, Netherlands; ^7^Research Department, Brain Innovation B.V., Maastricht, Netherlands; ^8^Department of Psychiatry and Psychotherapy, Charité Universitätsmedizin Berlin, Berlin, Germany; ^9^Department of Neuroscience, Imaging and Clinical Sciences, Institute for Advanced Biomedical Technologies, D'Annunzio University of Chieti–Pescara, Chieti, Italy; ^10^Neurology Department, University Hospital of Wales, Cardiff, United Kingdom; ^11^School of Health, Sport and Bioscience, University of East London, London, United Kingdom; ^12^Faculty of Health, Medicine and Life Sciences, School for Mental Health and Neuroscience, Maastricht University, Maastricht, Netherlands

**Keywords:** fMRI, neurofeedback, stroke, preregistration, rehabilitation

## Abstract

Ischemic stroke of the middle cerebral artery (MCA), a major brain vessel that supplies the primary motor and premotor cortex, is one of the most common causes for severe upper limb impairment. Currently available motor rehabilitation training largely lacks satisfying efficacy with over 70% of stroke survivors showing residual upper limb dysfunction. Motor imagery-based functional magnetic resonance imaging neurofeedback (fMRI-NF) has been suggested as a potential therapeutic technique to improve motor impairment in stroke survivors. In this preregistered proof-of-concept study (https://osf.io/y69jc/), we translated graded fMRI-NF training, a new paradigm that we have previously studied in healthy participants, to first-time MCA stroke survivors with residual mild to severe impairment of upper limb motor function. Neurofeedback was provided from the supplementary motor area (SMA) targeting two different neurofeedback target levels (low and high). We hypothesized that MCA stroke survivors will show (1) sustained SMA-region of interest (ROI) activation and (2) a difference in SMA-ROI activation between low and high neurofeedback conditions during graded fMRI-NF training. At the group level, we found only anecdotal evidence for these preregistered hypotheses. At the individual level, we found anecdotal to moderate evidence for the absence of the hypothesized graded effect for most subjects. These null findings are relevant for future attempts to employ fMRI-NF training in stroke survivors. The study introduces a Bayesian sequential sampling plan, which incorporates prior knowledge, yielding higher sensitivity. The sampling plan was preregistered together with *a priori* hypotheses and all planned analysis before data collection to address potential publication/researcher biases. Unforeseen difficulties in the translation of our paradigm to a clinical setting required some deviations from the preregistered protocol. We explicitly detail these changes, discuss the accompanied additional challenges that can arise in clinical neurofeedback studies, and formulate recommendations for how these can be addressed. Taken together, this work provides new insights about the feasibility of motor imagery-based graded fMRI-NF training in MCA stroke survivors and serves as a first example for comprehensive study preregistration of an (fMRI) neurofeedback experiment.

## Introduction

Ischemic stroke of the middle cerebral artery (MCA) is one of the most common forms of stroke (Leys et al., 1992; Aouad et al., 2013). The MCA is the main blood supply to the primary motor cortex, including the hand knob area, as well as the premotor cortex. Hence, MCA stroke often leads to severe upper limb impairment and compromises patients' quality of life (Miller et al., 2010; Langhorne et al., 2011). The efficacy of current rehabilitative strategies is limited, and most patients remain impaired such that more than 70% of stroke survivors show acute upper limb impairment (Lawrence et al., 2001) and in many cases to a moderate-to-severe degree. Furthermore, it is estimated that only 5–20% of patients with hemiparesis regain full upper limb function, while 33–60% do not show any recovery 6 months after stroke (Kwakkel and Kollen, 2013). This remaining limb dysfunction presents a major impediment to rehabilitation, activities of daily living and occupational prospects of stroke survivors, and has a considerable, negative effect on their well-being (Langhorne et al., 2011; Pollock et al., 2014). Hence, there is a need for new noninvasive therapies to promote recovery of motor function in general and in particular for the upper limb after MCA stroke.

Several motor imagery-based interventions have been suggested for stroke (Sharma et al., 2009b; Ietswaart et al., 2011), which may enhance neuroplasticity and thus potentially facilitate recovery, in particular when combined with regular physiotherapy (García Carrasco and Aboitiz Cantalapiedra, 2016; Sakurada et al., 2017). Such interventions include brain–computer interfaces (Cervera et al., 2018) and real-time fMRI neurofeedback training (Linden and Turner, 2016; Wang et al., 2017) whereby participants engage in mental practice to control some form of external feedback that they are provided with. Motor imagery strategies can be primarily visual or kinesthetic, and it is possible that the type of motor imagery influences the degree of improvement in motor impairment (Jackson et al., 2001; Sharma et al., 2009b). Whereas, visual motor imagery focuses primarily on visual mental imagery, either from a first- or a third-person perspective, kinesthetic motor imagery is defined as motor imagery from the first-person perspective and involves imagining the feeling and experience of movements without overt movement. Interestingly, brain stimulation (Stinear et al., 2006) and neuroimaging studies (Solodkin et al., 2004; Guillot et al., 2009; Hétu et al., 2013; Sharma and Baron, 2013) suggest that kinesthetic motor imagery recruits motor areas including the supplementary motor area (SMA) and that this type of imagery is thus of interest for motor rehabilitation. In a previous neurofeedback experiment conducted in healthy participants, we confirmed that the SMA is a suitable target for motor imagery-based neurofeedback training (Mehler et al., 2019b). Specifically, we have previously introduced a new form of feedback training where participants self-regulated the activity of the SMA to discrete target levels. In this within-subject design, users act as their control condition regarding self-regulation success of motor imagery-based neurofeedback training based on a measure that is calibrated by individuals' baseline activation during a localizer scan. In the present proof-of-concept (PoC) study, we aimed to translate these findings to MCA stroke patients.

We introduce two new aspects to the fMRI-neurofeedback community: comprehensive study preregistration and a Bayesian sequential sampling plan (Schönbrodt et al., 2015). Before data acquisition started, the study protocol was preregistered on an open platform (the Open Science Framework) to increase the replicability and reproducibility of the introduced methods and reported results. Adapted from clinical trial registrations (Nosek et al., 2018), our comprehensive study preregistration included a transparent description of the methodology, *a priori* hypotheses, and a detailed analysis plan outlining how it is intended to test for these (https://osf.io/y69jc/#!). Hence, comprehensive study preregistration address three common concerns in translational neuroscience and research more broadly (Poldrack et al., 2017; Mehler, 2019): Unanticipated challenges, which are part of developing a new intervention technique, are disclosed transparently in the process, affording an adequate discussion that can eventually help addressing these in future work. Moreover, predeclared analysis plans help preventing questionable research practices. Lastly, with preregistration results are published irrespective of their outcome, effectively addressing publication bias (Allen and Mehler, 2019). The preregistration protocol included a Bayesian sequential sampling, which allows accumulating evidence for (or against) an effect until a certain threshold is reached (Schönbrodt and Wagenmakers, 2018). Moreover, individual tests were calibrated (using a preregistered decision rule) by individual prior distributions. These were informed by the SMA activation that was measured at the beginning of a training session during a localizer scan. Such approach provides greater flexibility and sensitivity over conventional (frequentist) sampling plans and is a promising approach for translational research designs. The study's aim was mainly to test for the feasibility of graded fMRI neurofeedback training of the SMA in first time MCA stroke patients. The SMA is an attractive target region of interest (ROI) in MCA stroke patients because it is supplied by the anterior cerebral artery (Brugger et al., 2015) and thus is not expected to have (substantially) compromised hemodynamic function following MCA stroke. Our first hypothesis was that patients could activate the SMA through motor imagery. Our second hypothesis was that patients can self-regulate the feedback signal to two discrete target levels.

## Methods

### Preregistration Protocol

The following methods section is based on the preregistered study protocol (Mehler et al., 2017). Minor modifications were performed, partly based on reviewers' feedback, to improve the documentation.

### Participants

This PoC study aims to ascertain if unilateral MCA ischemic stroke patients, with moderate to severe upper limb impairment, are able to (1) achieve sustained SMA activation during motor imagery and (2) demonstrate control over the feedback (by self-regulating the activation to different target levels), which we see as prerequisites for the use of graded neurofeedback training as motor rehabilitation therapy. Stroke severity was determined by the local clinician and based on the Modified Rankin Scale Questionnaire Score of 3–4 (Bonita and Beaglehole, 1988). Potentially suitable participants, within 6 months of stroke, were identified by clinical staff at Cardiff and Vale University Health Board. The inclusion criteria for patients were as follows:

1. infarction of the middle cerebral artery territory
2. persisting hemiplegia/hemiparesis
3. patients either (a) have been discharged from the acute stroke unit and received ambulatory physiotherapy (so called *early supported discharge*), (b) are still undergoing, or (c) have already completed an inpatient rehabilitation program
4. no receptive aphasia to ensure that task instructions will be properly followed.

Patient exclusion criteria were any MRI contraindications. Patients provided informed consent. The study has been approved by the local research ethics committee (Wales REC3, reference number 16/WA/0167) operated by Health and Care Research Wales. For a detailed list of exclusion criteria that affect the data acquisition and/or quality, please see *Data Exclusion* section below. Although exclusions have been anticipated, any further expulsions and reasons are possible and will be documented (please see *Deviations From Preregistered Protocol*).

### Data Exclusion

Neurofeedback runs that contain too much head motion, defined by >30% volumes with a framewise displacement (FD) > 0.5 mm were excluded (Power et al., 2014).

Head motion was quantified using framewise displacement (FD), with:

(1) $$\begin{array}{r} FD_{\text{i}}=|\Delta d_{\text{ix}}|+|\Delta d_{\text{iy}}|+|\Delta d_{\text{iz}}|+|\Delta \alpha_{\text{i}}|+|\Delta \beta_{\text{i}}|+\;|\Delta_{\text{γ}i}|, \end{array}$$

where *i* indicates the volume, Δd_ix_ = d_(i−1)x_ − d_ix_ for translation rigid body motion parameters, and [α_i_ β_i_ γ_i_] are the three head rotation parameters (roll, pitch, yaw) that were converted to millimeters using a projection onto a sphere with a radius of 50 mm (Power et al., 2014).

If debriefing suggested that patients misunderstood instructions, data of affected runs were excluded. Furthermore, if patients did not attend the second neurofeedback session and did not withdraw their consent, acquired data from the first neurofeedback session that met the quality criteria as specified above were still included in the Sequential Bayes Factor sampling and all other planned analyses.

If technical problems occurred that either disrupted or delayed the feedback presentation, data from affected runs were excluded. Incomplete neurofeedback runs were excluded and repeated. If patients interrupted a scan session but consent was not withdrawn, acquired data from this session were included. However, some instances in which a patient stopped complying during the task were identified after debriefing, and those affected runs were excluded. Other unanticipated circumstances affecting data quality could result in exclusions. In such circumstances, all exclusions and reasons are reported (please see *Deviations From Preregistered Protocol*).

### Dependent Variables and Hypotheses

The first dependent variable of interest is the median percent signal change (PSC) of the SMA-ROI during motor imagery (taking an average across the low and high neurofeedback condition) > rest (details for calculation, see *Analysis Plan* below). The second dependent variable of interest is the difference in SMA-ROI PSC between the low and high neurofeedback condition (details for calculation, see *Analysis Plan* below).

H0_A_: MCA stroke patients show no sustained SMA-ROI activation.
H1_A_: MCA stroke patients will show sustained SMA-ROI activation as measured by a positive PSC.
H0_B_: MCA stroke patients show no difference in SMA-ROI activation (measured by PSC) between low and high neurofeedback conditions.
H1_B_: MCA stroke patients show a difference in SMA-ROI activation between low and high neurofeedback conditions.

### Sample Size and Bayesian Sequential Sampling Plan

Owing to feasibility and PoC, we followed a Bayesian sampling strategy. Bayesian statistics do not require adjustment for multiple testing and hence can provide higher flexibility and sensitivity over conventional (frequentist) sampling plans (Schönbrodt and Wagenmakers, 2018). Moreover, the interpretation of the Bayes factor, which expresses how much more likely one hypothesis is over another, allows sampling data until evidence for the absence of an effect is found. For the present study, we chose a minimum of *N* = 5 patients and continue recruiting either until the Bayes factor for both hypotheses (A and B) was conclusive—i.e., either for the alternative with a Bayes factor (BF_10_) > 10 (indicating that the alternative hypothesis is at least 10 times more likely than the null hypothesis given the data, thus indicating strong evidence for a positive effect) or for the null with BF_01_ > 10 (indicating that the null hypothesis is at least 10 times more likely than the alternative hypothesis given the data, thus indicating strong evidence for a null effect)—or until the end of the data collection period (February 28, 2018) was reached.

For both H1_A_ and H1_B_, Bayes factor calculations were performed using a normal prior (Baguley, 2010; Dienes, 2014). For H1_A_, we tested for activation vs. no activation and thus conducted a one-sided one-sample Bayesian *t* test for which the prior distribution was scaled by the group median of the PSC during the localizer (PSC_LOC_). For H1_B_, we tested for a difference between the low and high neurofeedback level and thus conducted a one-sided paired Bayesian *t*-test. In the optimal case, the difference would be 0.5 (see paragraph above). Hence, to test for H1_B_, prior scaling of the prior distribution was set to 0.5.

Besides these Bayesian *t*-tests that were used for the stopping rule, we also report respective Bayesian *t*-tests on group level with a default Jeffreys–Zellner–Siow (JZS) prior (Cauchy *r* = 0.707) (Rouder et al., 2009). These are reported together with the median of the posterior and its 95% credible interval. To test if resulting Bayes factors were sensitive to priors, *a prior* robustness check (with *r* = 0.5, *r* = 1, and *r* = 1.4142) was conducted to assess the robustness of the outcome (Rouder et al., 2016; Schönbrodt and Wagenmakers, 2018).

This stepwise Bayesian approach provides us with more flexibility to detect larger effects and hence to stop sampling earlier. It also allows flexible accumulation of evidence for the null hypothesis, which presents one main advantage of Bayesian hypothesis testing compared to conventional frequentist hypothesis tests (Mehler et al., 2019a). To control for early stopping due to false positives or false negatives, we have set a relatively conservative stopping threshold of BF = 10 for both the alternative and the null hypothesis. However, there remains a risk for inflated type-II error rates to detect small to medium effect sizes given the minimum *N* = 5. For instance, such sample would require at least an effect size of Cohen's *d* = 1.36 for the planned one-sided frequentist *t* tests to be powered at 80%. Hence, in line with best practice recommendations, we acknowledge this limitation by labeling the study proof of concept (Ros et al., 2020).

### Procedure

Patients were invited to two fMRI-NF training sessions. Sessions were conducted at the patient's convenience but within 6 months of their stroke. If necessary, patients were made familiar with the MR scanner environment using a mock scanner before their scanning session. Mental imagery performance has been shown repeatedly to be modulated by hand orientation (Jongsma et al., 2013) as well as body posture (de Lange et al., 2006; Ionta et al., 2012). Thus, patients were asked to identify a kinesthetic motor imagery strategy that involves both hands (see *Motor Imagery Instructions*).

### MRI Acquisition and Online Processing

Imaging data were acquired using a 3-T MRI scanner (3T Prisma, Siemens Healthcare, Erlangen, Germany) at the Cardiff University Brain Research Imaging Center (CUBRIC). Blood-oxygenation-level-dependent (BOLD) signals during localizer and neurofeedback runs (see *Procedure*) were measured with a T2^*^-weighted gradient-echo echo planar imaging (EPI) sequence synchronized to the onset of the stimulus presentation. Functional EPI volumes of 24 slices of 2.5-mm thickness, with 0.5-mm interslice spacing was used (in-plane resolution = 3 mm, TR = 1,500 ms, TE = 30 ms, flip angle = 80°). High-resolution structural images were acquired before the first functional scan using a magnetization-prepared rapid gradient-echo sequence (MPRAGE) T1-weighted image with 172 contiguous sagittal slices of 1-mm thickness (voxel size: 1 × 1 × 1 mm, TR = 7.9 s, TE = 3.0 ms, flip angle = 20°, FoV = 256 × 256 × 172 mm). Turbo-BrainVoyager (TBV) software (Brain Innovation B.V., Maastricht, Netherlands, version 3.2) was used for online preprocessing and analysis of BOLD signals including motion correction with respect to the first volume of the functional localizer and spatial smoothing [4 mm full width at half maximum (FWHM)].

### Functional Localizer

The functional localizer run (180 volumes) consisted of four blocks (30 s) kinesthetic motor imagery, flanked by rest blocks (30 s). An incremental general linear model (GLM) was used including a task predictor and linear drift term to compute task-correlated activity. The localizer run serves to identify most active voxels and to calculate the individual percent signal change (PSC_LOC_) that is used to scale the visual feedback.

Previous real-time neurofeedback studies with neurological patients have either used motor execution (Subramanian et al., 2011, 2016) or action observation (Sitaram et al., 2012) tasks during localizer runs. However, given that motor execution is impaired in stroke patients in general and to different degrees across patients, a motor execution localizer would be difficult to implement and most likely provide unreliable estimates for PSC_LOC_. Moreover, more recent studies that employed graded fMRI-NF also used mental imagery localizers (Sorger et al., 2016; Krause et al., 2017). Lastly, different subparts within the SMA may be activated during motor imagery compared to motor execution, and hence, a motor imagery-based localizer may be more suitable for imagery-based neurofeedback training (Mehler et al., 2019b). Statistically significant voxels were selected using a *t*-contrast of motor imagery > rest with a variable *t*-threshold to ensure that a sufficient number of voxels were available within the SMA for the following selection. The 40 most active neighboring voxels (determined by their *t* value) in four neighboring slices were identified in native functional space based on this *t*-contrast using a custom-made plugin (Best Voxel Tool with settings 4-4-1-0-40; plugin available upon request) (Lührs et al., 2017). This procedure deviated from the originally preregistered procedure in which this step was conducted in a normalized space and constrained to an anatomical mask of the SMA (see justification provided in *Deviations From Preregistered Protocol*). To give participants sufficient time to disengage from motor imagery during the rest and allow time for the BOLD signal to recover to baseline, only the second half of each rest period were considered for the calculation using a custom-made PSC plugin tool (*PSC calculation Tool*; plugin available upon request).

The PSC_LOC_ value determined the 100% level of the thermometer feedback; the remaining parts of the thermometer display are linearly scaled accordingly (see *Neurofeedback Calculation and Presentation*). Specifically, the PSC_LOC_ value to scale the thermometer was set to 80% of the maximum percent signal change measured in the localizer. However, in case patients initially struggled to engage in motor imagery when no feedback is provided yet, a lower bound PSC_LOC_ = 0.7 was set to avoid underestimating an appropriate PSC_LOC_ to scale the feedback. Furthermore, because the maximum PSC value could be biased by outliers or spikes in the time series, an upper bound was set to PSC_LOC_ = 1.4. These values are comparable to PSC_LOC_ default values of 1% that have previously been used in neurological patients (Subramanian et al., 2011, 2016).

### Neurofeedback Calculation and Presentation

The localizer run was followed by five neurofeedback runs (180 volumes), which contained two repetitions of two block types, a low and high neurofeedback level, which were interleaved by rest (30 s). For feedback presentation, the mean raw BOLD value was extracted from the SMA-ROI (BOLD_SMA−ROI_). The feedback was computed (temporally smoothed) based on Equation 2:

(2) $$\begin{array}{r} PSC_{NF}=\frac{\left(val-baseline\;\right)*100}{baseline} \end{array}$$

where *val* is the mean of three consecutive BOLD_SMA−ROI_ values, *baseline* is the median BOLD_SMA−ROI_ during the second half (i.e., last 10 TRs) of the preceding rest period, and PSC_NF is the resulting percent signal change. PSC_NF_ was then normalized by PSC_LOC_ to map it on to the (15) segments of the thermometer display such that every segment represents 10% of the PSC_LOC_. Values below 0 were rounded up to 0; values above 15 were rounded down to 15. The calculation was carried out using an in-house written python script (Python 2.7.10). The Open Source Python library Expyriment was used for continuous online feedback presentation (Krause and Lindemann, 2014). Target levels were defined by 50% (low) and 100% (high) of the localizer SMA-ROI PSC and indicated by green arrows. Both the low- and high-level conditions were repeated twice per run and interleaved by rest periods. During rest periods, no feedback was presented. The order of the condition (low and high target level) was counterbalanced across runs and subjects (determined by subject number and run number). The python script for the localizer and neurofeedback runs is available (https://osf.io/y69jc/).

### Motor Imagery Instructions

Before the scan, patients were asked to identify a kinesthetic motor imagery strategy that involved both hands and that they could perform comfortably, vividly, and consistently (e.g., an activity of daily living) for ~30 s while lying in a supine position comparable to the actual scan session. Patients were further instructed to avoid any movements and muscle contractions.

During the localizer run, patients were asked to use this motor imagery strategy while being presented with an empty thermometer on the screen. Patients were instructed to remain still in the scanner and either rest when presented with red arrows next to the empty thermometer gauge or perform vivid kinesthetic mental imagery of an action that involves both of their hands during task periods that were indicated with green arrows. Patients were reminded that no feedback was presented during the localizer scan.

For both the localizer and neurofeedback runs, patients were further instructed that, during scans, they should (1) remain still and relaxed, (2) avoid movements and muscle contractions, and (3) use only kinesthetic motor imagery that involves both hands. Besides these aspects, patients were not restricted in the content of motor imagery (e.g., a particular type of activity or sport).

For the neurofeedback runs, it was explained to patients that bars in the thermometer represent the activity level in the target region and that their goal is to use kinesthetic motor imagery to control the feedback by filling up the bars contained in the thermometer display. They were also instructed to maintain the activation at target levels by adjusting their mental strategy (e.g., changing speed and/or intensity of the imagined movement).

### Offline fMRI Analysis Plan

The present study is a repeated measure within-subject design. The dependent variable of interest is the PSC of the SMA-ROI in the contrast task—i.e., supervised kinesthetic motor imagery based on fMRI-NF from the SMA-ROI > rest. The open source software AFNI (version 16.2.18) was used for offline fMRI ROI analyses using the same preprocessing parameters (motion correction, 4 mm spatial smoothing) as used for online feedback. Based on existing in-house scripts, AFNI functions *3dDeconvolve* and *3dREMLfit* function were used. The AFNI function *3dDeconvolve* was used to calculate task and baseline predictors (see below), and preprocessed BOLD time series and design matrices were submitted to *3dREMLfit* to compute SMA-ROI PSCs. This approach largely replicates the analysis carried out online and additionally corrects for temporal autocorrelation (AR1) of the BOLD time series. For the functional localizer, the intercept, drift, and task are modeled, and the PSC is defined as the ratio between task and intercept parameter estimates. For the neurofeedback runs, analyses was carried out on a concatenated time series for both the low and high target level with their respective preceding rest periods such that five coefficient estimates are returned for: the (1) intercept, (2) low-level task block, (3) high-level task blocks, (4) baseline blocks preceding low-level task blocks, and (5) baseline blocks preceding high-level task blocks (with order of 2–5 depending on randomization). Based on these coefficient estimates, PSCs are calculated with Equation 3:

(3) $$\begin{array}{r} PSC=task\;*\frac{100}{intercept+preceding\;restperiod} \end{array}$$

for the low and high neurofeedback level, respectively.

Returned values from the neurofeedback runs were normalized by the offline calculated PSC_LOC_. Hence, for instance, a value of 0.5 would indicate that 50% of the PSC_LOC_ have been achieved during neurofeedback, the desirable target level for the low neurofeedback condition.

Both SMA activation (H1_A_) and difference between target levels (H1_B_) were tested on the group level as well as in individual patients. Group analyses were carried out based on patients' median PSC values calculated across all runs from both neurofeedback sessions. To test for H1_A_, the grand median was calculated from PSC (grand mdPSC) values across the low and high target level conditions. To test for H1_B_, the median PSC was calculated separately for the low and high target level condition (mdPSC). In addition to the group analyses, both H1_A_ (SMA activation) and H1_B_ (graded SMA activation) were tested within subjects based on all PSC values available for a patient.

### Frequentist Statistical Analysis

Besides Bayesian hypothesis testing, frequentist hypothesis testing was also conducted. H1_A_ was tested with a one-sample *t*-test and H1_B_ with a paired *t*-test for both the group analysis and individual subject analyses. Because we have directed hypotheses in the cases of H1_A_ and H1_B_ (i.e., positive differences), all tests were carried out right tailed. Both hypotheses were tested on the group level as well as in individual patients. Tests conducted on group level were carried out at a significance level of 0.05. Tests at subject level were Bonferroni corrected for multiple comparisons and carried out at a significance level of 0.05/number of tested patients. For all measures, effects sizes (Cohen's *d*) are reported.

### Deviations From Preregistered Protocol and Exploratory Analyses

To address challenges in the data acquisition and analysis that were not anticipated when this study was preregistered (Mehler et al., 2017), the following changes to the preregistered methodology were applied.

#### Participants and Sessions

The patient recruitment was slower than expected. We therefore extended the recruitment phase by 5 months with approval from the ethics and research governance committees from September 30, 2017 until February 28, 2018. Toward the end of the recruitment phase, inclusion criteria (in terms of clinical severity) were relaxed to reach the minimum *N* = 5. As a result, one mildly impaired (patient 4; P4) and one recovered patient (P5) were included. In anticipation of potential data loss due to head motion, data from three training sessions were recorded for P3. P5 only completed one training session since the end date of the study was reached.

#### Coregistration and Functional Localizer

**Figure 1:**
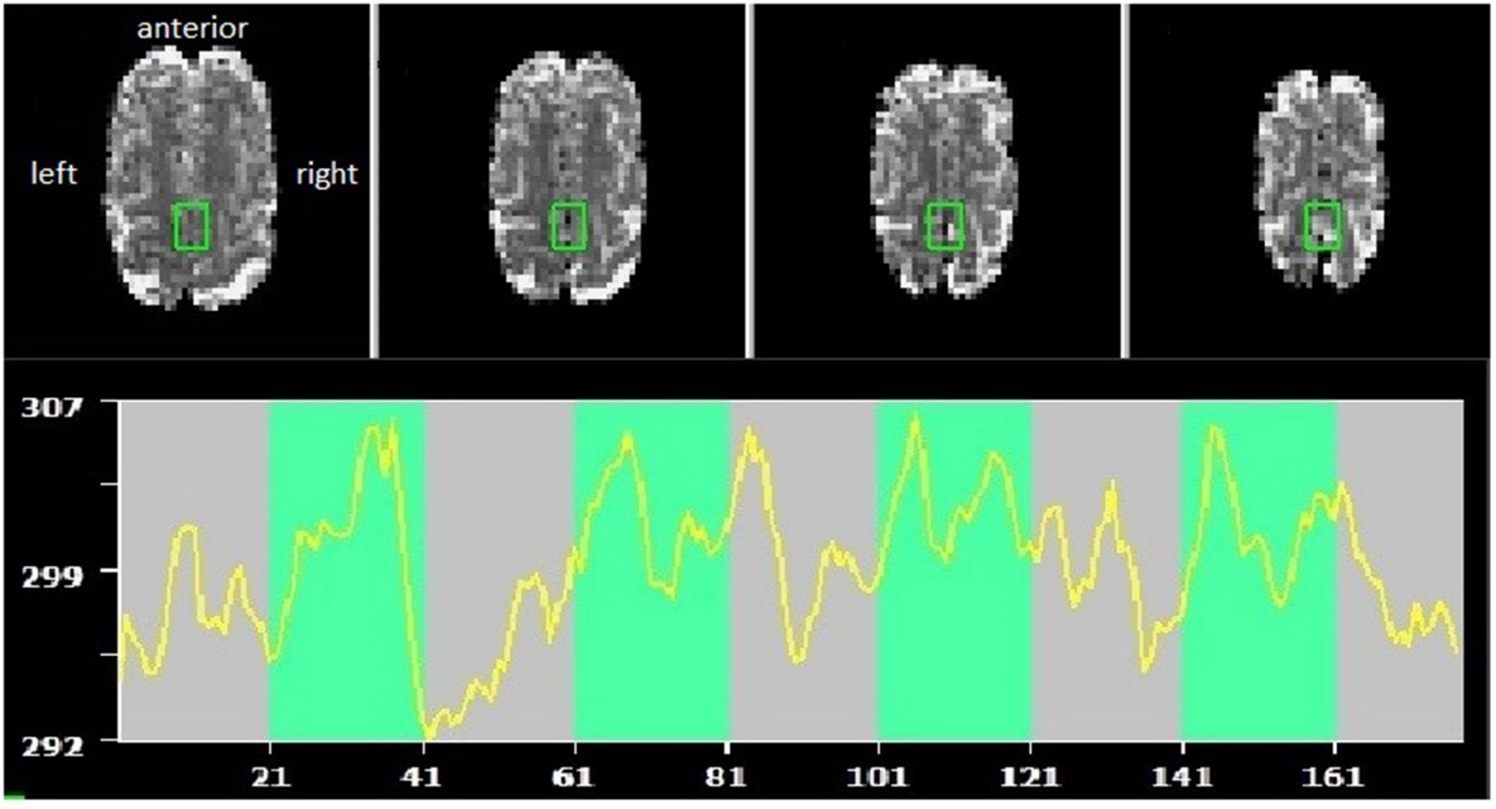
Example of supplementary motor area (SMA) target region of interest (ROI) and self-regulation time course (green indicating the task periods, red rest periods). Radiological convention, with anterior part of the brain shown at the top.

Brain lesions and head motion corrupted data quality and anatomical landmarks. As a consequence, planned online coregistration attempts failed. Therefore, the created anatomical SMA mask in Talairach could not be applied, and instead, target voxel selection was guided by visual inspection of functional brain slices in native space. For an exemplary target ROI and time course, see Figure 1.

Target regions were mostly constrained to the 40 most active adjacent voxels; however, this approach failed for two scanning sessions due to technical problems and resulted in a larger set of voxels (84 and 440) selected. Originally, it was planned to guide voxel selection using a custom-made SMA-ROI. For a detailed description about how the customized template was created, please see Supplementary Material 1).

#### Prior Distribution

In the original analysis plan, Bayesian analyses were conducted using a uniform prior distribution using custom-written MATLAB (Mathworks Inc.) scripts. However, to enhance reproducibility, it was decided to switch to the open source software JASP (version 0.8.4.0; Team, 2017), where users can set priors based on normal distributions. These were scaled based on localizer PSC values as described in the preregistered methods.

#### Mental Imagery Questionnaire

Patients evaluated their motor imagery capacity through a standardized motor imagery questionnaire, the Kinesthetic and Visual Imagery Questionnaire (KVIQ). The KVIQ includes test items for visual and kinesthetic motor imagery for the left and right upper limb, respectively, which are rated on a scale from 1 to 5 (Malouin et al., 2007). The questionnaire was used to familiarize patients with motor imagery, as well as to evaluate patient's self-ratings. For this study, only items 3–5 of the upper limb section were assessed, which included motor imagery tasks concerning the shoulder, elbow, and fingers. Scores for each body side and modality were calculated, such that a maximum of 15 points could be scored.

## Results

### Recruitment

**Figure 2:**
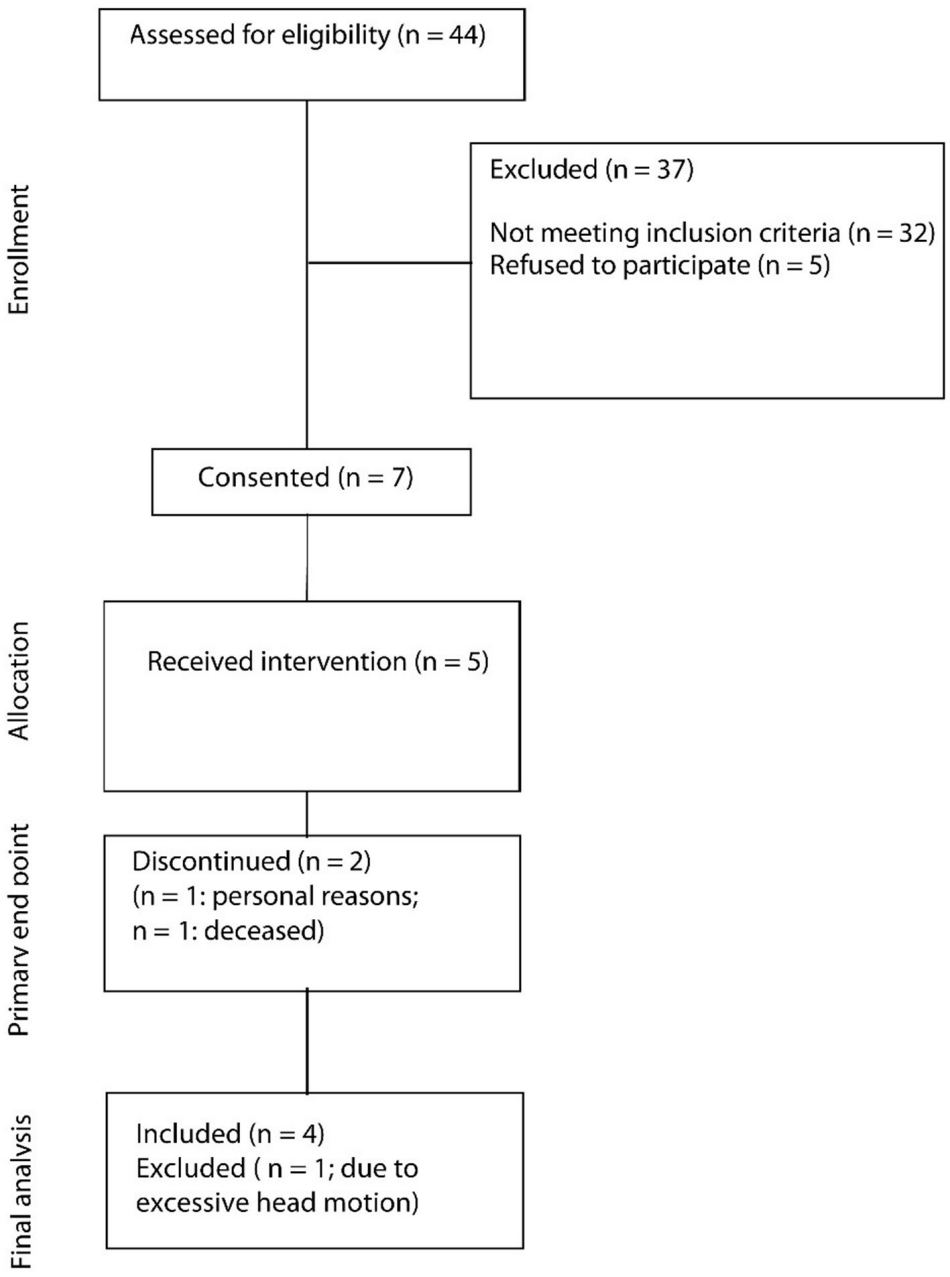
CONSORT recruitment flow diagram.

During the recruitment period (December 1st, 2016–February 28, 2018), 44 patients were screened for their eligibility (Figure 2). In total, 37 patients (84%) had to be excluded because they did not meet inclusion criteria. Leading reasons were overall poor health conditions (13 patients, 30%), either as a complication of the stroke or due to pre-existing comorbidities, as well as cognitive impairment (12 patients, 27%). Another five patients (11%) declined to participate for personal reasons. In total, seven patients (16%) provided consent to be included in the study, of which five patients completed the study (11%). One patient withdrew consent later due to personal reasons, and one patient died before starting the study.

### Patient Demographics

The patients that completed the study mainly suffered from right hemispheric MCA stroke, resulting in left-sided hemiparesis (Table 1). Overall, patients were relatively young, with a median age of 49 years (range, 38–68 years), broadly gender balanced (three female and two male patients), and mostly identified themselves as right-handed (*N* = 4; one left-handed). Patients further mostly suffered from right-hemispheric MCA stroke (four right hemispheric stroke, one left- hemispheric stroke).

**Table 1:** Patient demographics, stroke characteristics, motor impairment, and average head motion (±SD) during real-time fMRI neurofeedback training.

**Patient**	**Lesion side**	**Fugl meyer (severity)**	**Median ± interquartile range/Median percentage of excluded scan volumes**
P1	R	42/66 (moderate)	0.26 ± 0.13 mm/ 14.5%
P2^&^	R	13/66 (very severe)	1.19 ± 0.03 mm/ 97.8%
P3	R	43/66 (moderate)	0.27 ± 0.08 mm/ 6.7%
P4^*^	R	66/66 (fully recovered)	0.32 ± 0.11 mm/ 14.5%
P5*	L	52/66 (mild)	0.17 ± 0.02/ 1.1%

* *Patients 4 and 5 were recruited during the extended period when initial inclusion criteria were relaxed*.

& *Patient 2 was excluded from data analysis due to excessive head motion*.

### Preregistered Analyses

After fMRI data preprocessing and motion correction were performed, data were excluded based on the head motion criteria described above. Specifically, all data acquired from patient 2 (P2), as well as two runs from patient 1 (P1), were excluded because of severe head motion. In total, ~33% of all acquired volumes contained head motion above the set threshold of 0.5 mm. The exclusion of entire runs when more than 30% of volumes passed this threshold resulted in discarding 15 of 50 (30%) acquired functional imaging runs. The groups' median localizer PSC value was 1.12. However, in three sessions, the lower boundary value of 0.7 was used because the PSC estimation procedure yielded a value that was too low.

**Figure 3:**
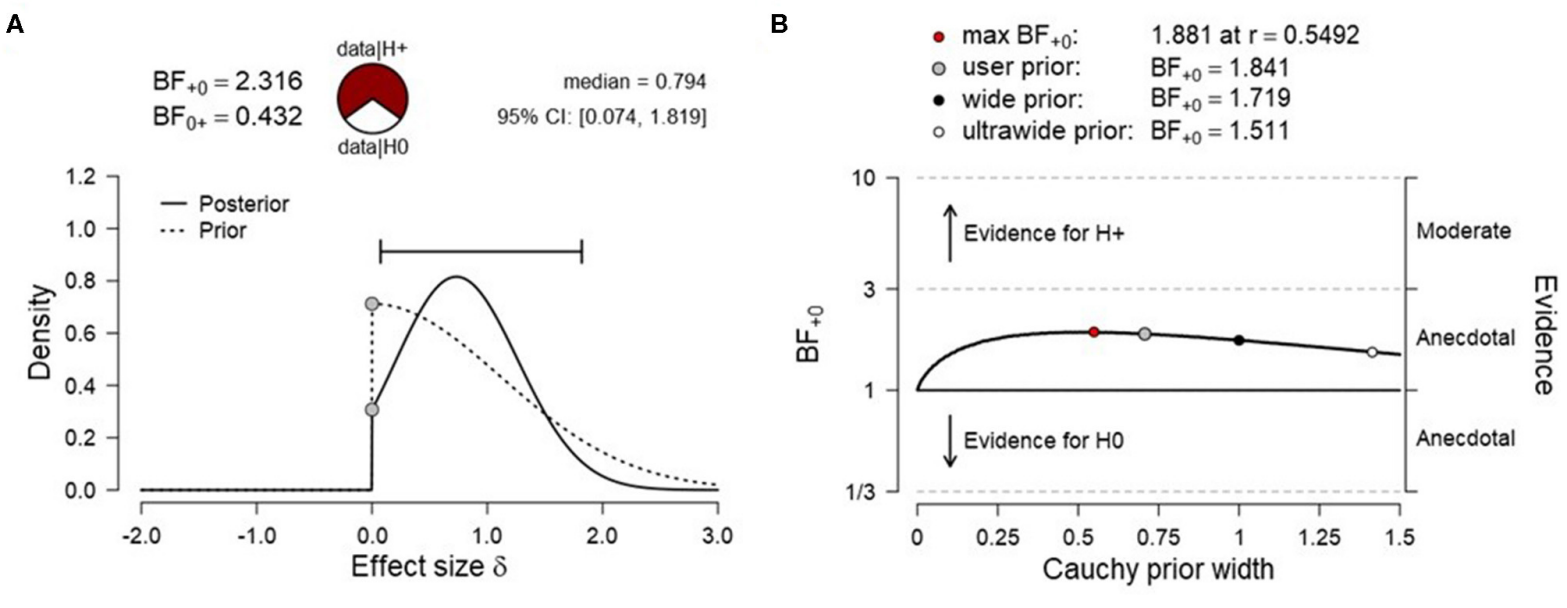
**(A)** Prior and posterior distribution for Bayesian *t* test and associated Bayes factor and effect size estimates for H1_A_ [net activation of the supplementary motor area (SMA)]. **(B)** Corresponding prior sensitivity analysis for a set of default Cauchy prior scaling settings.

To test H1_A_ (net activation of the SMA), data were submitted to a right-tailed one-sample Bayesian *t* test. The results showed only anecdotal evidence for SMA activation during fMRI-NF blocks compared to rest [N (0, 1.12), BF_+0_ = 2.316, median posterior Cohen's *d* = 0.794, 95% credibility interval (95% CrI) (0.074, 1.819), Figure 3A], indicating that the alternative hypothesis (H1_A_) was ~2.3 times more likely than the null hypothesis (H0_A_). The prior sensitivity analysis that was carried out with JZS priors and a range of scaling factors were overall comparable and suggested anecdotal evidence for SMA activation (Figure 3B). This convergence between analyses suggested that the results were relatively invariant to the prior distribution being used (Rouder et al., 2016). Data were also tested with a right-tailed one-sample frequentist *t*-test, which remained inconclusive [*t*_3_ = 1.789, *p* = 0.086, mean difference to zero of 0.142, 95% CrI (−0.045, ∞), Cohen's *d* = 0.895, 95% CrI (−0.161, ∞)]. Given the small sample, assumptions of normality of the data were tested. A Shapiro–Wilk test indicated that these were met (*W* = 0.808, *p* = 0.118).

**Figure 4:**
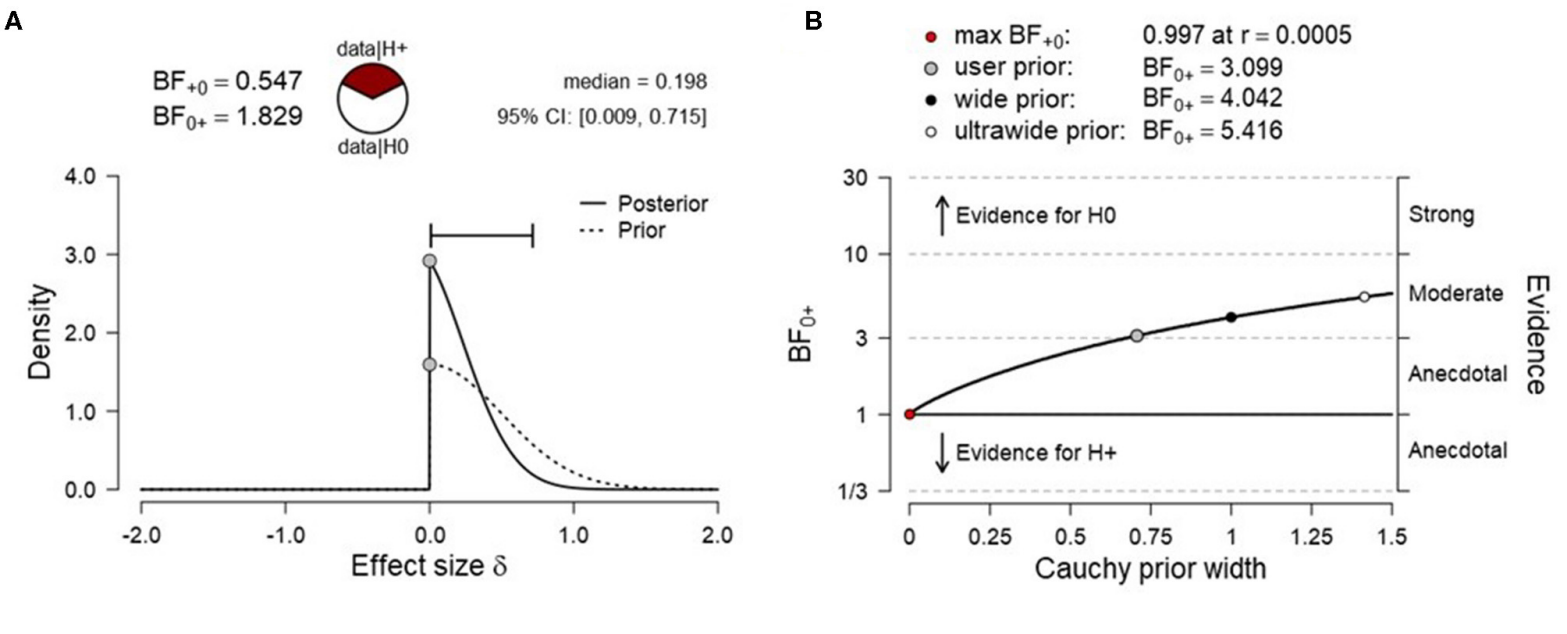
**(A)** Prior and posterior distribution for Bayesian *t* test and associated Bayes factor and effect size estimates for H1_B_ [more supplementary motor area (SMA) activation for the high vs. low target level condition]. **(B)** Corresponding prior sensitivity analysis for a set of default Cauchy prior scaling settings.

To test H1_B_ (more SMA activation for high vs. low target level condition), data were submitted to a right-tailed paired Bayesian *t*-test. The results showed anecdotal evidence for the absence of a level effect [N(0, 0.5), BF_0+_ = 1.829, median posterior Cohen's *d* = −0.198, 95% CrI (−0.009, −0.715), Figure 4A], indicating that the null hypothesis (H0_B_) was ~1.8 times more likely than the alternative hypothesis (H1_B_). The prior sensitivity analysis suggested moderate evidence for SMA activation for various scaling factors and the evidence for the null increased monotonically with increasing prior width (Figure 4B). A frequentist right-tailed *t*-test remained inconclusive [*t*_3_ = −0.483, *p* = 0.669, mean difference = −0.014, 95% CI (–.083, ∞), Cohen's *d* = −0.242, 95% CI (−1.06, ∞)].

Next, H1_A_ and H1_B_ were tested on subject level using frequentist *t* tests. A test for non-normality indicated no deviation for any patient with regards to their average PSC values (Supplementary Tables 1, 2). To test for net SMA activation, one-sided one-sample *t*-tests were conducted for patients individually. Tests for P1 and P3 remained inconclusive, and for P1, there was a trend opposite to the expected direction (SMA deactivation). Tests for P4 and P5 reached significance when uncorrected; however, when corrected for multiple testing using Bonferroni, only the effect for P5 remained significant (Table 2).

**Table 2:** Frequentist one sample *t* tests (H1_A_) on subject level.

				**95% CI for location parameter**			**95% CI for effect size**	
	***t***	***p***	**Location parameter**	**Lower**	**Upper**	**Effect size**	**Lower**	**Upper**
P1	−0.42	0.653	−0.03	−0.191	∞	−0.189	−0.921	∞
P3	1.34	0.126	0.04	−0.024	∞	0.599	−0.241	∞
P4	2.598	0.030	0.153	0.028	∞	1.162	0.130	∞
P5	5.488	0.003^*^	0.401	−0.116	∞	2.454	0.827	∞

* *Significant at Bonferroni corrected alpha = 0.0125*.

Bayesian one-sample *t*-tests that were informed by patients' individual activation during the motor imagery localizer scan indicated moderate evidence for P4 and P5 (Table 3). Noteworthy, these two patients also showed the least motor impairment (Table 1).

**Table 3:** Bayesian one-sample *t* tests (H1_A_) on subject level.

	**BF_**+0**_**
P1	0.525
P3	1.759
P4	3.579
P5	6.906

Paired *t-*tests for high vs. low target level remained inconclusive for P1, P3, and P4, with mean differences indicating a trend opposite to the expected direction (i.e., PSC_low_ > PSC_high_). Tests were only significant for P5 before, but not after correction for multiple testing (Table 4).

**Table 4:** Frequentist paired sample *t* tests (H1_B_) on subject level.

					**95% CI for Cohen's** ***d***		
	***t***	***p***	**Mean difference**	**SE difference**	**Cohen's *d***	**Lower**	**Upper**
P1	−0.75	0.753	−0.047	0.062	−0.336	−1.076	∞
P3	−0.92	0.794	−0.066	0.072	−0.409	−0.156	∞
P4	−2.22	0.949	−0.060	0.029	−0.946	−1.809	∞
P5	2.53	0.032	0.057	0.022	1.132	0.122	∞

Bayesian *t*-tests suggested anecdotal to moderate evidence for an absence of a level effect for P1, P3, and P4, and moderate evidence for the presence of a target level effect for P5 (Table 5).

**Table 5:** Bayesian paired samples *t* tests (H1_B_) on subject level.

	**BF_**0+**_**
P1	2.201
P3	2.337
P4	3.009
P5	0.288

Taken together, data suggested anecdotal or moderate evidence for SMA activation (H1_A_) in two patients, respectively. With regards to level effects, one patient showed moderate evidence for an effect (H1_B_), whereas the remaining patients showed anecdotal to moderate evidence for the absence of an effect (H0_B_). PSC data and JASP analysis files are available (https://osf.io/y69jc/).

### Exploratory Analyses

The following analyses were not predeclared in the preregistration protocol and served to explore hypotheses that were declared post hoc.

#### Self-Regulation Over Time

**Figure 5:**
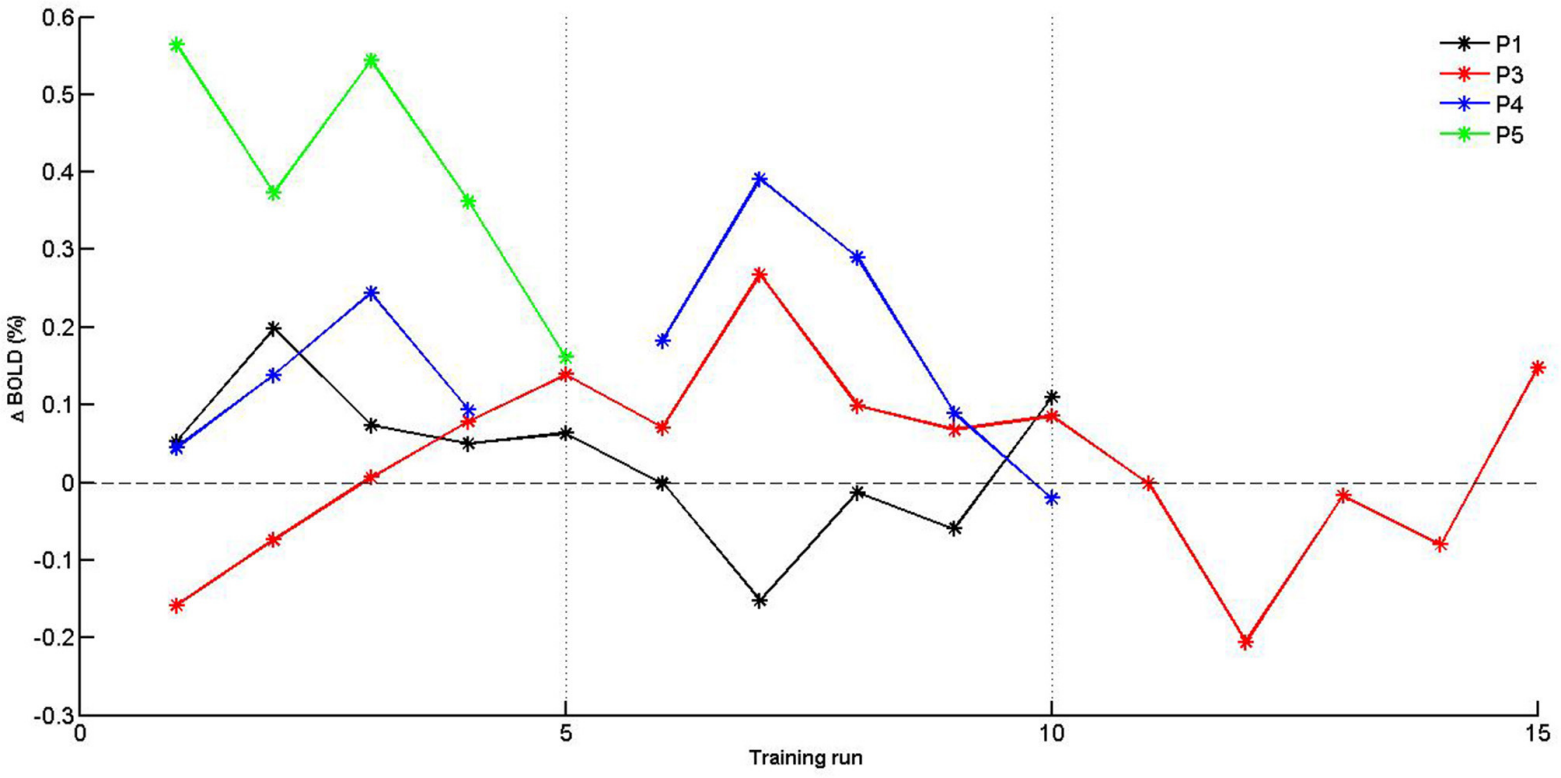
Individual PSC (Δ BOLD) values of supplementary motor area (SMA) activation for training runs. Separate training sessions indicated by dotted vertical lines.

**Figure 6:**
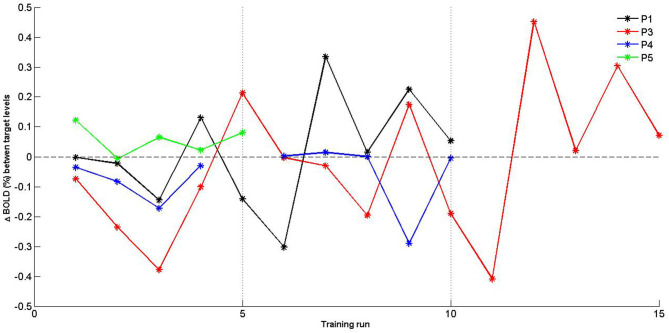
Individual percent signal change (PSC) (Δ BOLD) values of supplementary motor area (SMA) target level separation for training runs. Separate training sessions indicated by dotted vertical lines.

Given the limited sample size, self-regulation data were descriptively explored. PSC values were plotted for SMA activation (H1_A_), and target level separation (H1_B_) were plotted for individual subjects over time (i.e., training runs and sessions). With regards to SMA activation (Figure 5), we report two observations: first, data did mostly not indicate that the SMA was more activated over training runs within training sessions, except for session of P3 that showed a linear trend during session 1. Second, for two of three patients (P3 and P4) for which data from multiple sessions were available, data indicated increased activation between training sessions. With regards SMA target level separation (Figure 6), neither a trend within nor between training sessions was obvious for any of the patients.

#### Head Motion

Head motion-based data exclusion criteria indicated that about one-third (33.02%) of the acquired volumes contained head motion beyond the predefined thresholds. This data exclusion compromises significantly the power of the current study, in particular in combination with a second threshold that discards entire runs if more than 30% of volumes are affected.

For comparison, we applied the same threshold (FD > 0.5 mm) to existing head motion data acquired from a previous experiment with a similar fMRI-NF paradigm that was conducted in young healthy participants (Mehler et al., 2019b). We found that only ~3.4% of volumes showed too large motion (compared to 33% for patients in the present study) and that median head motion ranged from 0.10 to 0.25 mm between participants with a group median of 0.15 mm (compared to 0.17–1.19 mm between patients with a group median of 0.32 mm for the present study). Hence, head motion in our sample of stroke patients was substantially larger.

#### Relation Between Brain Lesion Side and Motor Imagery Ratings

To evaluate differences in self-rated motor imagery obtained from the KIVQ (Malouin et al., 2007), scores obtained of the affected side were subtracted from the scores of the non-affected side. Positive scores thus reflect a higher rating for the non-affected bodyside, an outcome that one may expect based on reports of motor imagery in stroke patients (Malouin et al., 2007; McInnes et al., 2016). Patients rated their motor imagery ability as being relatively high (for both visual and kinesthetic imagery), with no discernible difference between the affected and unaffected sides (Table 6). Likewise, no clear relationship with respect to patients' handedness was evident (e.g., ratings for right motor imagery were mostly not higher for patients who self-identified as right dominant).

**Table 6:** Patients' side of dominance and stroke, self-rated motor imagery sum scores for visual imagery (VI), and kinesthetic imagery (KI) for the left and right upper limb (out of 15 possible points, with higher values denoting higher imagery capacity).

**Patient**	**Dominant body side**	**Affected body side**	**VI left**	**VI right**	**VI Diff**	**KI left**	**KI right**	**KI Diff**
P1	L	L	5	4	−1	7	7	0
P2	R	L	10	9	−1	12	9	−3
P3	R	L	12	8	−4	15	15	0
P4	R	L	6	7	1	10	10	0
P5	R	R	9	9	0	13	9	4

Difference scores are calculated with respect to the non-affected side. Positive difference scores reflect larger ratings for the non-affected side; negative difference scores reflect larger ratings for the affected side.

## Discussion

Analyses from this preregistered PoC study found only anecdotal evidence for the two main preregistered hypotheses, suggesting that the present sample of MCA stroke patients struggled to activate and self-regulate the SMA during kinesthetic motor imagery-based graded neurofeedback training. Comparing individual effect sizes and Bayes factors (Tables 2, 3) found for SMA activation (H1_A_) with patients' motor impairment scores (Table 1), we found that patients who were the least impaired (P4 and P5) showed the largest SMA activation. Such potential relationship should be tested in future, larger controlled studies. Moreover, descriptive/visual data exploration of self-regulation values suggested that some patients (P3 and P4) may learn to increase SMA activation (H1_A_) over training sessions (Figure 5). Based on our limited experience, however, we note that, in particular, more severely impaired patients may not tolerate longer or additional training sessions well. The merely marginal evidence for SMA activation that we found overall during motor imagery may be surprising in light of earlier reports of SMA activation during motor imagery in chronic stroke patients (Sharma et al., 2009a,b; Confalonieri et al., 2012; Sharma and Baron, 2013). Besides larger sample sizes of earlier reports, differences in patient selection procedures may explain these discrepancies.

For instance, Sharma and colleagues used a rigorous screening procedure to assess motor imagery abilities at baseline (Sharma et al., 2009a,b). Thereby, these studies ascertained that included patients were skilled in performing motor imagery but at the cost of excluding 30–50% of patients (Simmons et al., 2008; Sharma et al., 2009a,b). Intriguingly, screening for baseline motor imagery capacity may also explain why samples of previous motor imagery studies featured mainly, or even exclusively, left hemispheric stroke patients (Sharma et al., 2009a), who tend to show less severe impairment in motor imagery tasks (Malouin et al., 2012) compared to right hemispheric stroke patients (Kemlin et al., 2016). In contrast, the present study mostly included right hemispheric stroke patients. Damage to the frontoparietal network has been hypothesized as a potential pathophysiological mechanism underlying these imagery deficits (Buch et al., 2012; Dettmers et al., 2015). For instance, the network is involved in cognitive processes that involve spatial attention and body–environment interactions (Ptak, 2012), functions that are likely involved in different forms of motor imagery. Although the present study used a motor imagery questionnaire that has been validated in stroke patients to assess self-rated motor imagery capacities (Malouin et al., 2007), it was not used to select patients based on their ratings. The main aim of this PoC study was to test the technique within the constraints of an established healthcare setting to provide a proxy measure of feasibility with an ecologically meaningful sample. Moreover, given the limited recruitment success, further restriction on patients would have additionally decreased the statistical power and biased the sample toward patients with above average motor imagery capacities. Taken together, the presented imaging findings are limited given the small sample size that has likely compromised the statistical power and also the fact that motor imagery abilities might have been impaired in patients due to mostly right-hemispheric stroke.

### Limitations

Patients were young with a median age of 49 years, a sampling bias that may have resulted from only including patients with sufficient cognitive capacities and general health who were more likely to complete the intervention (Sreedharan et al., 2019). Furthermore, as noted earlier, the sample mainly consisted of patients suffering from right-hemispheric stroke. Such bias may have resulted from excluding aphasic patients (who usually suffer from left hemispheric stroke). Hence, the generalizability of presented findings is likely compromised.

It is possible that providing feedback based on the BOLD signal may itself be a limitation. The BOLD contrast depends on coupling relationships between the metabolic rate of oxygen (CMRO_2_), cerebral blood flow (CBF), and volume (CBV). Changes in any of these parameters (e.g., due to inflammation) may attenuate BOLD responses. Indeed, reduced neurovascular coupling (Schroeter et al., 2007; Lin et al., 2011) and deficits in increasing local perfusion (Siegel et al., 2017) are well documented for stroke patients. Such pathological changes likely result in atypical BOLD dynamics in stroke patients and thus render interpretation of the present findings, i.e., only marginal evidence found for SMA activation, difficult. We therefore recommend that future fMRI-neurofeedback studies should include neurovascular measurements such as breath-hold functional scans that allow assessing vascular reactivity in stroke patients (Murphy et al., 2011; Geranmayeh et al., 2015), as well as CBF and CBV weighted data that can help understanding null findings related to the BOLD signal (Blicher et al., 2012).

Lastly, the present study only focused on testing whether patients were able to activate the SMA and gain control over it, rather than behavioral effects of the training. Hence, no control group was included, also in order to increase the number of patients that could be recruited to test for feasibility. Future studies will benefit from including behavioral outcome measures and pre–post assessments, possibly including a treatment as usual group that is matched for motor impairment and age (see for review, Sorger et al., 2019) or a an active control group as previously employed in randomized clinical trials (RCTs) of fMRI-NF for other conditions (Subramanian et al., 2016; Mehler et al., 2018) and fNIRS-NF training (Mihara et al., 2013).

### Challenges and Potential Solutions

Three main challenges were encountered in the present study: (1) recruiting a sufficient number of eligible patients, (2) substantial head motion, and (3) additional efforts that resulted from preregistering the protocol *a priori*.

(1) The present sample of five patients was recruited out of 44 screened patients; the main reason to exclude patients was impaired cognitive capacity and lack of general well-being. As a consequence, the trial period was extended by 5 months and inclusion criteria relaxed to reach the minimum target of five patients, which is comparable to sample sizes of previous PoC fMRI-NF studies in neurological populations (Subramanian et al., 2011; Sitaram et al., 2012; Liew et al., 2015; Sreedharan et al., 2019). Multicenter studies with standardized protocols and less rigorous inclusion/exclusion criteria will likely accelerate recruitment. Alternatively, technologies that are more portable and comfortable such as functional near-infrared spectroscopy (fNIRS) (Kohl et al., 2019) and electroencephalography, which have been successfully tested in stroke (Mihara et al., 2013; Lioi et al., 2020), may yield higher recruitment rates and attainment compared to fMRI. Furthermore, researchers may benefit from preregistering (Bayesian) sequential sampling plans, such as introduced here. These allow flexible stopping rules that are informed by prior knowledge and calibrating statistical tests based on patient characteristics, thereby increasing design efficiency.

(2) Another factor that compromised the data was head motion. Although head motion poses a major challenge for fMRI research with stroke patients (Siegel et al., 2017) and fMRI-neurofeedback more generally (Heunis et al., 2020), several previous fMRI studies that investigated motor imagery stroke patients have not reported criteria to control for it (Sharma et al., 2009b; Sharma and Baron, 2013; Liew et al., 2015). The present study employed rigorous criteria informed by previous recommendations (Power et al., 2012, 2014; Siegel et al., 2017) leading to a substantial discarding of data (an increase by the factor of nearly 10 compared to a similar study conducted in healthy participants) and one patient from the analysis. Noteworthy, head motion in stroke may relate to impairment severity, for instance due to general discomfort as reported by P2 (who showed consistently large head motion and was excluded from the analysis), and thus, data censoring may increase the risk of sampling bias (Wylie et al., 2014). Preferably, future work should make use of more recently developed real-time correction procedures and quality control tools to reduce head motion (Maclaren et al., 2013; Dosenbach et al., 2017; Heunis et al., 2019; Krause et al., 2019). Lastly, neurofeedback technologies such as fNIRS, where the signal acquisition is more resilient toward head motion, may provide a useful alternative (Kohl et al., 2019).

(3) To address concerns around replicability in neuroimaging (Poldrack et al., 2017; Mehler, 2019) and follow best practice recommendations (Ros et al., 2020), the present study was preregistered. This approach not only protects against publication and researcher biases (Algermissen and Mehler, 2018) but also comes with additional challenges (Allen and Mehler, 2019). For instance, in order to formulate an analysis plan, *a priori* knowledge about plausible effects and various contingencies is needed. Noteworthy, this approach still allows exploring data to generate hypotheses about underlying mechanisms; however, data exploration was very limited in the present study due to the small sample size and data quality issues. To document the design and reporting quality, we have completed the “Consensus on the reporting and experimental design of clinical and cognitive-behavioral neurofeedback studies (CRED-nf checklist)” (Ros et al., 2020) and made results available (https://osf.io/p46xb/). The present work was based on a previous study (Mehler et al., 2019b), which allowed us to adapt an existing paradigm to stroke patients more easily (for instance, a motor execution localizer was replaced by a motor imagery localizer). However, we note that researchers may not always have the opportunity to test a paradigm in a healthy population first. Moreover, we underestimated recruitment difficulties and data quality issues due to distortions and head motion, which required slight deviations from the original protocol. For instance, MRI coregistration failed at setup, mainly because anatomical image quality was insufficient. The preregistration of his PoC study allowed us to document transparently where we had to deviate from our originally intended design, providing a more realistic picture of the challenges that come with this type of research. We thus encourage researchers to preregister their studies and consider predeclaring analysis plans that are contingent on achieved sample sizes for populations that are difficult to recruit and where statistical power requirements may not be met. For instance, such preregistration could include a minimum sample size that is required to employ inferential statistics and otherwise mainly draw conclusions based on data description.

Lastly, we note that the SMA remains an attractive target region not for motor imagery-based interventions not only in stroke but also other motor conditions (Hampson et al., 2011; Sukhodolsky et al., 2019). As an alternative to providing feedback based on an average signal from an entire ROI, future fMRI-NF studies may benefit from exploring connectome-based (Ramot et al., 2017; Noble et al., 2020) and multivariate, i.e., decoded, neurofeedback (Shibata et al., 2019). Connectome-based neurofeedback allows targeting entire networks of correlated activity between nodes of motor networks and support their reorganization (Desowska and Turner, 2019), allowing to probe causally functional network models of impairment/recovery (Mehler and Kording, 2018). Of particular interest for future work may be targeting dynamic connectivity states that vary with motor impairment (Bonkhoff et al., 2020). Multivariate approach yield higher sensitivity compared to mass univariate models. These allow detecting neural correlates of imagined (Zabicki et al., 2016) and executed movements (Ejaz et al., 2015; Kolasinski et al., 2020) down to the level of individual fingers (Oblak et al., 2020). Albeit promising, both approaches are highly susceptible to spurious correlations resulting from excessive head motion. Successful implementation in neurological populations will thus afford using reliable real-time head motion and artifact correction methods (Heunis et al., 2019, 2020).

## Conclusion

The present PoC study tested for the feasibility of motor imagery-based graded fMRI-NF neurofeedback training in a small sample of MCA stroke patients. Results suggested only anecdotal evidence for preregistered hypotheses, and replication in larger samples is required. To our knowledge, this is the first fully preregistered report of a neurofeedback study and the first neurofeedback study that employed a Bayesian sampling plan. Difficulties in recruiting eligible patients, adequate control for patient's head motion, a compromised BOLD signal, and the trade-off between applying adequate inclusion/exclusion criteria vs. inducing sampling biases should be considered in future fMRI-NF studies conducted with stroke patients.

## Data Availability Statement

The datasets generated for this study are available on request to the corresponding author.

## Ethics Statement

The studies involving human participants were reviewed and approved by Health and Care Research Wales, REC3, reference number 16/WA/0167. The patients/participants provided their written informed consent to participate in this study.

## Author Contributions

DM, AW, JW, FK, ML, RW, HS, DT, and DL have planned the study. DM performed motor assessments, analyzed all data, and drafted the manuscript. DM, JW, FK, and ML have implemented the setup. DM and SK have collected the imaging data. DM, HS, and DL performed patient screening and enroment. All authors have reviewed, commented and edited the manuscript.

## Conflict of Interest

ML was an employee of Brain Innovation B.V., the company that develops the Turbo-Brainvoyager software used for the real-time fMRI analyses. FK was a former employee of Brain Innovation B.V. The remaining authors declare that the research was conducted in the absence of any commercial or financial relationships that could be construed as a potential conflict of interest.
