# Supplementary material for: Graded fMRI Neurofeedback Training of Motor Imagery in Middle Cerebral Artery Stroke Patients: A Preregistered Proof-of-Concept Study
Source: Front Hum Neurosci. 2020 Jul 14;14:226. doi: 10.3389/fnhum.2020.00226 (PMC7373077; doi:10.3389/fnhum.2020.00226)
Supplement: Supplementary file 1 [file Table_1.DOCX]

Supplementary Material

# Supplementary Tables

**Table 1 Test of normality (Shapiro-Wilk) for H1_A_ on subject level based on average PSC values.**

|  | | **W** | | **p** | |
| --- | --- | --- | --- | --- | --- |
| P1 |  | 0.904 |  | 0.435 |  |
| P3 |  | 0.917 |  | 0.513 |  |
| P4 |  | 0.907 |  | 0.452 |  |
| P5 |  | 0.909 |  | 0.463 |  |

**Table 2 Test of Normality (Shapiro-Wilk) for H1_B_ on subject level based on differences of PSC values between target levels.**

|  | | **W** | | **p** | |
| --- | --- | --- | --- | --- | --- |
| P1 high - low |  | 0.973 |  | 0.892 |  |
| P3 high - low |  | 0.886 |  | 0.337 |  |
| P4 high - low |  | 0.883 |  | 0.322 |  |
| P5 high - low |  | 0.981 |  | 0.940 |  |

**2 Supplementary Material**

**SMA-ROI mask**

The original protocol aimed to conduct neurofeedback training in standard space and constrain the target region selection with an anatomical mask. However, due to technical challenges arising from problematic data quality, this online procedure failed. Since the created mask may be still of value for future work, it is provided on the OSF page (https://osf.io/y69jc/). Further, since importing a customized mask in Turbo-Brainvoyager (version 3.2) was not trivial and required a customized workflow that can be used for other purposes, it is documented in detail here. To create an SMA-ROI mask, an atlas template (Automated Anatomical labelling; AAL) (Tzourio-Mazoyer et al., 2002) was extracted from the PickAtlas tool (Maldjian et al., 2003, 2004). Further, the corresponding T1-weighted image (i.e. collin27) that these structures have been co-registered to was extracted from the SPM Anatomy toolbox (Eickhoff et al., 2005). The resulting NIfTI images require conversion into a data format that is readable by TBV. Because the conversion via the Brainvoyager NIfTI-1 converter plugin (v1.09) requires identical image dimensions (i.e. bounding boxes), the SMA-ROI was first be sampled to the T1-weighted image using Nilearn (version 0.3.0; resample_to_img function) (Abraham et al., 2014) and then converted to BrainVoyager anatomical format. Next, images were transformed from MNI to Talairach space using BrainVoyager (BrainInnovation, Maastricht, The Netherlands, version 20.4). Lastly, the obtained anatomical file was transformed into an SMA-ROI and thresholded (at minimum value range = 50) using Brainvoyager for use in Turbo-BrainVoyager.

**Supplementary References**

Abraham, A., Pedregosa, F., Eickenberg, M., Gervais, P., Mueller, A., Kossaifi, J., et al. (2014). Machine learning for neuroimaging with scikit-learn. *Front. Neuroinform.* 8, 14. doi:10.3389/fninf.2014.00014.

Eickhoff, S. B., Stephan, K. E., Mohlberg, H., Grefkes, C., Fink, G. R., Amunts, K., et al. (2005). A new SPM toolbox for combining probabilistic cytoarchitectonic maps and functional imaging data. *Neuroimage* 25, 1325–1335. doi:10.1016/j.neuroimage.2004.12.034.

Maldjian, J. A., Laurienti, P. J., and Burdette, J. H. (2004). Precentral gyrus discrepancy in electronic versions of the Talairach atlas. *Neuroimage* 21, 450–5. Available at: http://www.ncbi.nlm.nih.gov/pubmed/14741682 [Accessed April 26, 2017].

Maldjian, J. A., Laurienti, P. J., Kraft, R. A., and Burdette, J. H. (2003). An automated method for neuroanatomic and cytoarchitectonic atlas-based interrogation of fMRI data sets. *Neuroimage* 19, 1233–9. Available at: http://www.ncbi.nlm.nih.gov/pubmed/12880848 [Accessed April 26, 2017].

Tzourio-Mazoyer, N., Landeau, B., Papathanassiou, D., Crivello, F., Etard, O., Delcroix, N., et al. (2002). Automated Anatomical Labeling of Activations in SPM Using a Macroscopic Anatomical Parcellation of the MNI MRI Single-Subject Brain. doi:10.1006/nimg.2001.0978.
